# How do patients and other members of the public engage with the orphan drug development? A narrative qualitative synthesis

**DOI:** 10.1186/s13023-023-02682-w

**Published:** 2023-04-17

**Authors:** Julia Frost, Abi Hall, Emily Taylor, Sarah Lines, Jessica Mandizha, Catherine Pope

**Affiliations:** 1 College of Medicine and Health, University of Exeter, Exeter, EX1 2LU UK; 2 South West Peninsular ILD Service, Royal Devon University Healthcare NHS Foundation Trust, Barrack Road, Exeter, EX2 5DW UK; 3 Nuffield Department of Primary Care Health Sciences, University of Oxford, Radcliffe Observatory Quarter, Woodstock Road, Oxford, OX2 6GG UK

**Keywords:** Rare diseases, Orphan drugs, Patient engagement, Patient involvement, Qualitative research, Systematic review

## Abstract

**Background:**

The diversity of patient experiences of orphan drug development has until recently been overlooked, with the existing literature reporting the experience of some patients and not others. The current evidence base (the best available current research) is dominated by quantitative surveys and patient reported outcome measures defined by researchers. Where research that uses qualitative methods of data collection and analysis has been conducted, patient experiences have been studied using content analysis and automatic textual analysis, rather than in-depth qualitative analytical methods. Systematic reviews of patient engagement in orphan drug development have also excluded qualitative studies. The aim of this paper is to review qualitative literature about how patients and other members of the public engage with orphan drug development.

**Methods:**

We conducted a systematic search of qualitative papers describing a range of patient engagement practices and experiences were identified and screened. Included papers were appraised using a validated tool (CASP), supplemented by reporting guidance (COREQ), by two independent researchers.

**Results:**

262 papers were identified. Thirteen papers reported a range of methods of qualitative data collection. Many conflated patient and public involvement and engagement (PPIE) with qualitative research. Patients were typically recruited via their physician or patient organisations. We identified an absence of overarching philosophical or methodological frameworks, limited details of informed consent processes, and an absence of recognisable methods of data analysis. Our narrative synthesis suggests that patients and caregivers need to be involved in all aspects of trial design, including the selection of clinical endpoints that capture a wider range of outcomes, the identification of means to widen access to trial participation, the development of patient facing materials to optimise their decision making, and patients included in the dissemination of trial results.

**Conclusions:**

This narrative qualitative synthesis identified the explicit need for methodological rigour in research with patients with rare diseases (e.g. appropriate and innovative use of qualitative methods or PPIE, rather than their conflation); strenuous efforts to capture the perspectives of under-served, under-researched or seldom listened to communities with experience of rare diseases (e.g. creative recruitment and wider adoption of post-colonial practices); and a re-alignment of the research agenda (e.g. the use of co-design to enable patients to set the agenda, rather than respond to what they are being offered).

**Supplementary Information:**

The online version contains supplementary material available at 10.1186/s13023-023-02682-w.

## Background

Orphan diseases are often so rare that physicians have little knowledge of these conditions, while the contribution of patients to orphan drug development is under-documented [1]. Recent changes in the policy and regulatory landscape have enshrined the contribution of patients and patient organisations into the drug development lifecycle. On the 31st January 2022, the Clinical Trials Directive (EC) No. 2001/20/EC was repealed [2], and a transition period (until 2023) entered under Clinical Trials Regulation (Regulation (EU) No 536/2014) [3]. The objective of the new Clinical Trials Regulation is to harmonise the processes for assessment and supervision of clinical trials throughout the European Union (EU). The directive stipulates a greater role for patients and patient organisations (defined by the European Medicines Agency as not-for profit organisations which are patient focused, and whereby patients and/or carers -the latter when patients are unable to represent themselves- represent a majority of members in governing bodies) in the oversight of, and access to, clinical trials. In the UK, the Rare Disease Framework similarly outlined ambitions to improve patient access to new therapeutics, premised on a commitment to consultation with patient representatives, and explicitly those from Black, Asian and minority ethnic (BAME) or disadvantaged backgrounds [4].

Existing research suggests that the diversity of patient experiences of orphan drug trials has been overlooked [5, 6]. The current evidence base is dominated by surveys of patients who have had a positive experience of trial participation or treatment, practitioners who provide these treatments, or surveys of patient and public representatives interested in drug trial development [7, 8]. Where qualitative evidence of patient experiences have been collected, they have been subject to content analysis and automatic textual analysis [9, 10], rather than in-depth qualitative analyses that could inform improvement. There is a gap in knowledge around the experience of patients who have limited access to clinical trials of genomic treatments, and those who do not receive the active treatment, who withdraw from the trial, or for whom there is a perception that the drug is ineffective [11].

Recently, Brown and Bahri have proposed a conceptual and methodological framework for evaluating patient and public engagement in relation to pharmacovigilance, which delineated engagement in terms of three dimensions [12]:

- *Breadth*: the diversity of patient engagement;
- *Depth:* The extent of knowledge exchange between stakeholders; and
- *Texture:* The interactive dynamics of what engagement feels like, means to people, and shapes their motivations to engage and change behaviour-based on values, emotions, (mis)trusts, and rationales.

Furthermore, they note that qualitative research is particularly suited to evaluating both the perspectives and mechanisms of engagement activities. Noting a rise in the volume of quantitative research that purports to concern patient and public engagement in the orphan drug development lifecycle [13, 14], we employed Brown and Bahri’s framework to establish the extent to which corresponding qualitative research could deepen our understanding of current engagement practices [12].

The aim of this paper is to explore how patients and other members of the public engage with the process of orphan drug development.

## Methods

### Patient advisory group

A Patient Advisory Group (PAG) were convened prior to the funding application, and met regularly to discuss the scope and content of the research. The group consisted of 6 local members of a rare disease group, and 2 members of a national group. The scope and content of the review were also discussed with the Steering Group, which also includes a patient from an international patient organisation. The PAG did not want to be cited as authors [13] but we acknowledge their contribution to this review.

### Literature search

Orphan drug terminology is highly specialised, and we started our search by gathering a selection of papers using search methods that do not rely on keyword terminology. This was informed by the work of Zhao [14], who outlines the role of ‘meta-’ in the synthetic process, and the need to identify the ‘state of play’ of a given area of study. We followed Zhao’s advice to use qualitative synthesis *as diagnostic.* For Zhao: “[synthesis] starts with an examination of problems encountered in primary study and ends with prescriptions for resolving these” (14: 381).

To this end, we conducted forward citation searches of two topically relevant systematic reviews using Google Scholar (https://scholar.google.com/), which although had excluded qualitative papers during searching and screening exposed us to topically relevant literature [15, 16]. The lead author (JF) knew the systematic reviews from background reading. We also inspected the studies included in these reviews. Qualitative primary studies which met our inclusion criteria, and which could inform the development of the bibliographic database search strategy, were examined for keyword terminology. We also examined quantitative primary studies for topic related terminology, even though these would not be included within the analysis.

**Fig. 1:**
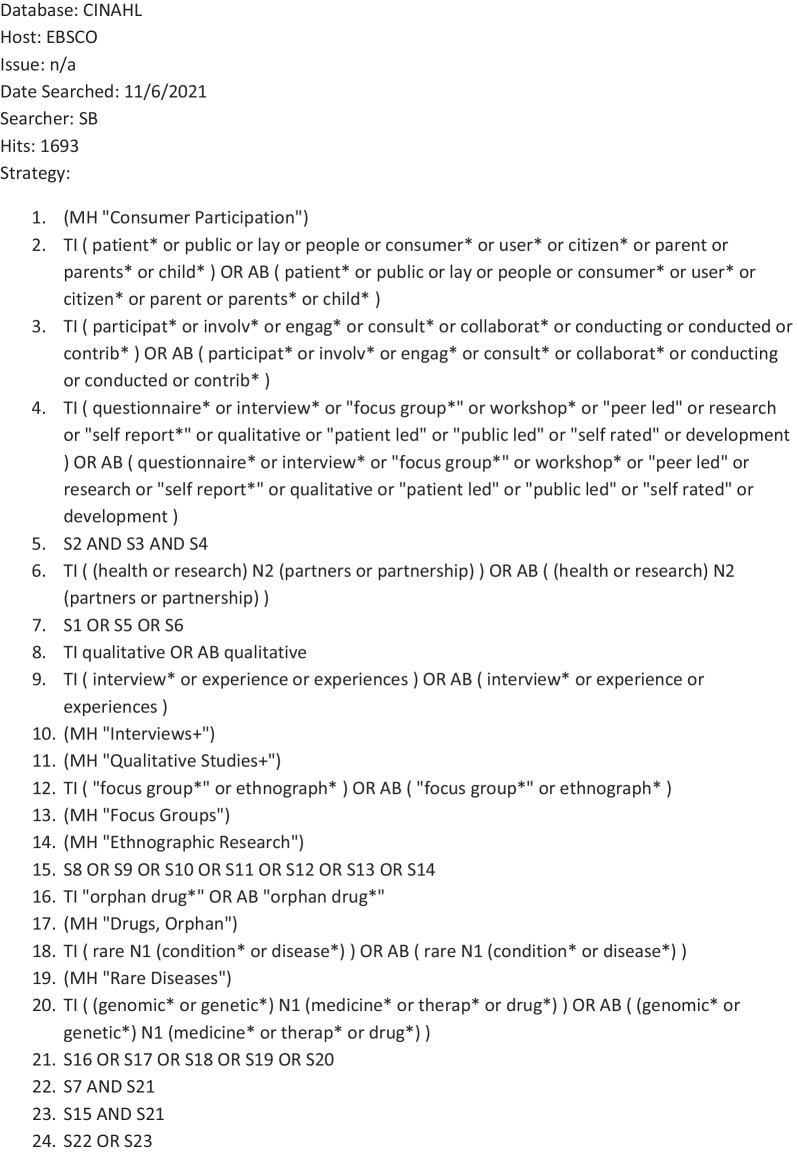

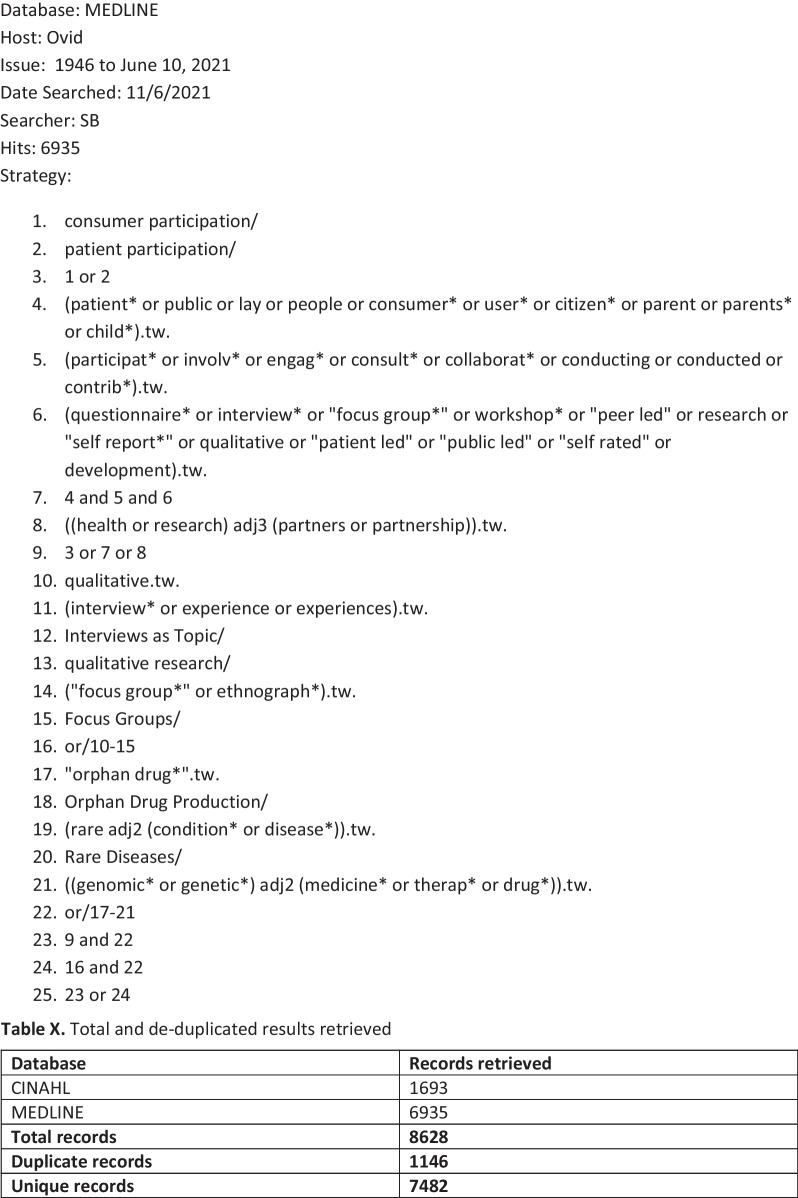
Orphan drugs search report

**Fig. 2:**
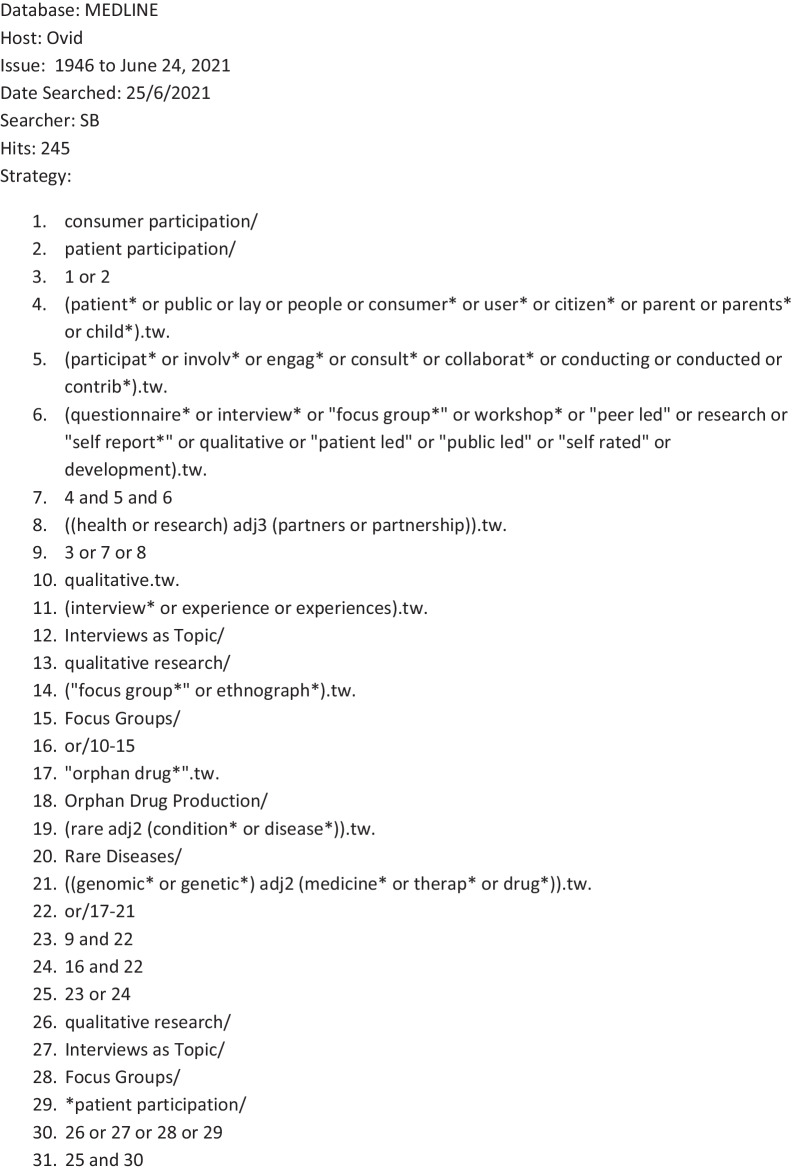
Amended search

The bibliographic database search strategy was developed by an information specialist in conjunction with the review team. The search strategy was developed in MEDLINE (via Ovid)). Search terms for orphan drugs and rare diseases were derived from the titles, abstracts and indexing terms (e.g. MeSH in MEDLINE) of pre-identified relevant studies and supplemented with appropriate synonyms. As a corrective to previous reviews, which had excluded qualitative papers, we combined these terms with two published search filters: a patient and public involvement search filter [17] and a qualitative search filter [18]. However this yielded a prohibitive number to screen in full (*n* = 6935), as many of the studies were irrelevant (e.g. beyond our area and scope of interest, Fig. 1: Initial search). We therefore focused the search by limiting the results to articles which were indexed with any of four highly discriminating methodological MeSH terms: qualitative research, interviews, focus groups, and patient participation. This retrieved a more focused sample (*n* = 262; Fig. 2: Amended search).. Our intention was not to conduct an exhaustive survey of the field, but to establish the extent to which qualitative research could deepen our understanding of current engagement practices. To do this does not require a review of all papers for all rare diseases, but instead draws on established qualitative sampling approaches, seeking ‘information power’; which depends on (a) the aim of the study, (b) sample specificity, (c) use of established theory, (d) quality of dialogue, and (e) analysis strategy [19]. As we wanted to sample a selection of relevant studies rather than search exhaustively, we limited the search to the MEDLINE database and the results of forward citation searching. The results of both the forward citation searches and the MEDLINE search were exported to Endnote X8 [20] and de-duplicated using both the automated de-duplication function and manual checking. The bibliographic database search was conducted on 11th June 2021.

### Quality appraisal

Two researchers (AH, ET), independently applied the Critical Appraisal Skills Programme (CASP) checklist to assess the quality of the studies selected for inclusion [21] (Table 1). Quality appraisal is contentious in qualitative syntheses, because there is limited consensus about what makes a study good [22–24]. To further understand the context in which the research was conducted, we also used a validated 32-item checklist to provide a means to assess the rigour and validity of the data collection and analysis techniques used by the research authors [25].

**Table 1:** Quality appraisal of included papers

	1. Clear statement of aims?	2. Appropriate methodology?	3. Research design appropriate?	4. Recruitment strategy appropriate	5. Data collection address the research issue?	6. Relationship between researcher and participant?	7. Ethical issues taken into consideration?	8. Data Analysis sufficiently rigorous?	9. Is there a clear statement of findings?	10. Value of the research
Bendixen et al. [33]	Y	Y	Y	Y	Y	N	Y	CNT	CNT	Y
Carroll et al. [7]	Y	Y	Y	Y	Y	N	Y	CNT	CNT	Y
Gaasterland et al. [42]	Y	Y	N	N	CNT	Y	CNT	CNT	CNT	Y
Gaasterland et al. [43]	Y	CNT	Y	Y	Y	N	Y	CNT	CNT	Y
Gengler [35]	Y	Y	Y	Y	Y	Y	CNT	.Y	Y	Y
Kesselheim et al. [36]	Y	Y	Y	Y	CNT	N	CNT	CNT	Y	Y
Lopes et al. [44]	Y	Y	Y	N	N	N	Y	N	N	Y
Li et al. [45]	Y	Y	Y	N	N	N	Y	N	N	Y
Menon et al. [38]	Y	Y	Y	Y	Y	N	Y	N	CNT	Y
Peay et al. [37]	Y	Y	Y	Y	Y	N	CNT	N	CNT	Y
Tingley et al. [39]	Y	Y	Y	CNT	Y	N	Y	Y	Y	Y
Tingley et al. [40]	Y	Y	Y	CNT	Y	N	Y	Y	Y	Y
Young et al. [41]	Y	Y	Y	CNT	Y	N	Y	Y	CNT	Y

Key: Y, Yes; N, No; CNT could not tell

### Synthesis method

The purpose of qualitative synthesis is to achieve greater understanding and attain a level of conceptual or theoretical development beyond that achieved in any individual empirical study [26]. We had planned to undertake a meta-ethnography [27], to identify where similar concepts and themes from different studies or papers refer to the same entity or to opposing findings, with the objective of moving current debates about patient engagement forward [14]. However, as the included papers identified were not deemed to be ‘conceptually rich’ in that they did not extend our understanding [28, 29]. A paper is considered to be conceptually rich in qualitative synthesis if it makes a substantial contribution to the synthesis. In this context, critical appraisal is not undertaken to exclude papers prior to the synthesis, but to ‘test’ the contributions of the papers at a later stage [30]. We therefore undertook a narrative synthesis, appropriate when a wide range of research designs are included, and to tell the story of existing data [31]. The lead author (JF) tabulated data from the included papers using a standardised data extraction table, which enabled the derivation of themes that mapped onto the lifecycle of orphan drug development [32], and these were discussed and refined by the wider research team.

## Results

**Fig. 3:**
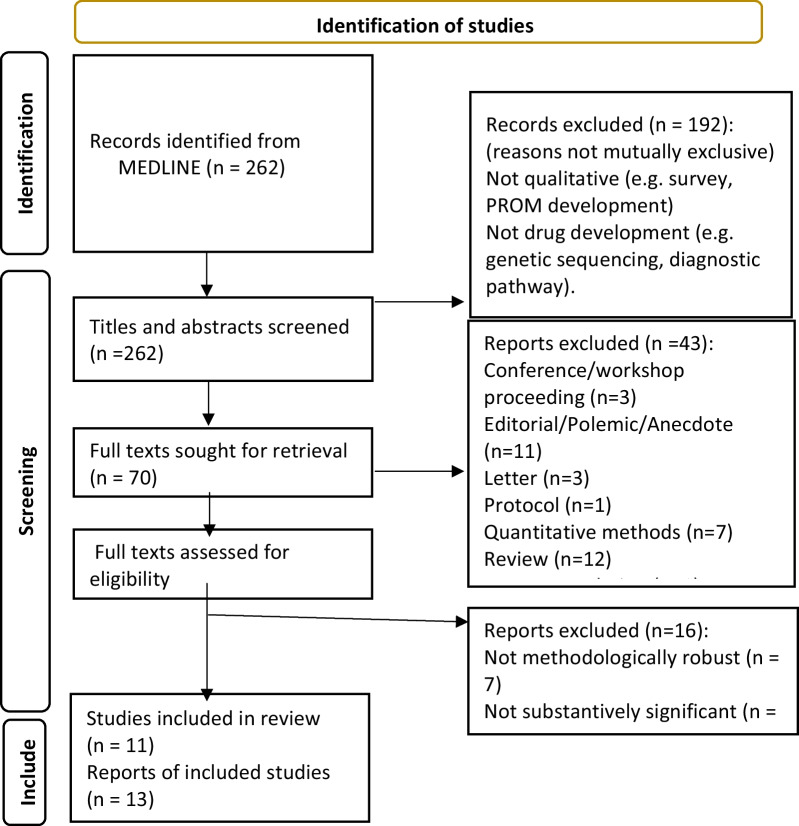
Identification of studies. *Adapted from:* Page MJ, McKenzie JE, Bossuyt PM, Boutron I, Hoffmann TC, Mulrow CD, et al. The PRISMA 2020 statement: an updated guideline for reporting systematic reviews. BMJ 2021;372:n71. https://doi.org/10.1136/bmj.n71. For more information, visit: http://www.prisma-statement.org/

Of the 262 abstracts identified, we excluded 192 that were explicitly quantitative (e.g. surveys, or concerning the development of patient reported outcome measures), or not about drug development (e.g. genetic sequencing and diagnostic pathways). Of the 70 full-text papers that we reviewed (Fig. 3: Identification of studies, and Additional file 1: Full texts retrieved), we excluded 57 papers that did not contain primary qualitative data (e.g. literature reviews, research protocols, opinion pieces, letters, editorials and organisations statements); were not deemed to be methodologically robust (e.g. they did not have sufficient information concerning recruitment, data collection or analysis to be replicable); or which were substantively not significant (e.g. they reported on focus groups or workshops, but the perspectives of rare disease patients, caregivers, representatives of patient organisations, or members of the public were missing, or could not be disaggregated from a wider ‘stakeholder voice’ that included health professionals or policy makers).

### Study characteristics

We included 13 published papers from a 10 year period (2012–2022)—with studies originating from the USA (5) [33–37], Canada (4) [38–41], Europe (2) [42, 43], Brazil (1) [44] and China (1) [45], and which detailed 11 separate studies (e.g. both papers by Gaasterland et al. [42, 43] and both papers by Tingley et al. [39, 40] pertained to the same data sources) (Table 2). Three papers recruited the parents of children with rare diseases who considered trials as a means to access treatment [33, 34, 36]; while two papers included representatives of patient organisations concerned by the lack of access to research in specific countries [44, 45]. The included papers formed a dataset spanning key junctures of the orphan drug development lifecycle.

**Table 2:** Study characteristics

References	Country	Aim	Recruitment/sample	Ethical approval	Data collection	Data analysis	Implications for widening engagement in orphan drug development
Bendixen et al. [33]	USA	To investigate *family-centred* and physician-based *attitudes and perceptions, to improve the future design* of Duchenne Muscular Dystrophy *clinical research protocols and improve participation in future clinical trials*	Sites involved in the Cooperative International Neuromuscular Research Group, a clinical research academic consortium, and associated Muscular Dystrophy Association clinics. 5 geographically and demographically diverse sites (Pittsburgh, Pennsylvania; Washington, DC; Houston, Texas; Minneapolis, Minnesota; and Sacramento, California) with varying levels of DMD research recruitment and participation were selected to maximize variability. e.g. sites varied from being active and engaged in numerous clinical studies in DMD to having had limited research involvement. Recruitment was focused on parents (primary care- givers) of boys with DMD. The inclusion criteria for parents: were limited to having a child with DMD, the ability to understand and speak English, and a willingness to participate in a 1-time-only focus-group interview lasting up to 120 min	Institutional Review Board, University of Pittsburgh (14010024). Written informed consent was obtained from all parents and clinicians before participating in this study	*28 parents* and 33 clinicians completed the 13 focus group sessions Focus-group sessions were conducted between July 2014 and October 2014 at each local site in central locations convenient to the participants. Parents in the DMD focus groups received $25 remuneration for participation and reimbursement for transportation. All participants received a free meal and the option of child care during the session	Thematic analysis was used to identify patterns of meaning across the datasets (Braun et al. 2006). Patterns within the datasets were identified through a rigorous approach of familiarization with the data through reading and rereading each transcription by 3 researchers (Shenton t al 2004). The dataset was then hand-coded line by line to identify important features of the data. Patterns within the data were synthesized and reduced to themes (Boeije 2010). Data relevant to each theme were reviewed and discussed at biweekly data-analysis meetings. Themes were refined and rephrased to describe the story being told by the parents and clinicians. We emailed each parent and clinician participant and provided a table of the thematic findings. Each participant was given the opportunity to provide feedback or comments 6 of 28 parents responded with affirmation and appreciation that their participation was beneficial	*Information:* There were issue of an over-abundance of information that was fragmented, difficult to obtain, or difficult to understand The approach of using registries for recruitment requires individuals to log on to a website and provide their (child’s) medical history, typically without any contact with clinical staff. This passive strategy may be ideal for highly motivated or informed volunteers who are specifically interested in research Recruitment opportunities for minorities are limited due to a belief that they will be non-compliant or unable to fulfil the research objectives *Conversation:* The importance of regular communication, follow-up with potential participants, and accessibility to research staff was evident *Barriers:* Burdensome travel commitments, especially with a disabled child in an unfamiliar city Not meeting the inclusion criteria for enrolment *Incentives:* Importance of providing rewards and incentives for the child participants. Acknowledgement that the participants are giving their time *Solutions:* Use of peer support, social media, and educational outreach
Carroll et al. [7]	USA	To understand the *motivations of patients* with Pulmonary Arterial Hypertension *for participating in RCTs* so as *to facilitate enrollment in future trials* among patients with similar diseases	Participants recruited from the Pulmonary Vascular Disease Program at the University of Pennsylvania	Institutional Review Board of the University of Pennsylvania (810,120)	Semi-structured interviews with *26 participants*, using a vignette of a hypothetical trial. Interviews (lasted 10–20 min)	Thematic data analysis and constant comparison techniques (Strauss and Corbin 1998; Ryan 2003)	*Medical Benefits:* Participants expressed hope that participation in RCTs of novel therapies for PAH would result in personal benefit *Medical risks of harm:* Participants were concerned about the side effects of experimental drugs, and consequences of forgoing their usual treatments *Non-medical benefits:* altruism; compensation/reimbursement *Non-medical burdens:* travel to and from study appointments
Gaasterland et al. [42]	Europe	“We present the POWER-tool (an acronym of *Patient participation in Outcome measure Weighting* for Rare diseases).”	Asterix Patient Think Tank: *10 patients* who had been educated about clinical research (patient representatives in the area of (rare)cancers, Duchenne Muscular Dystrophy (DMD), Mucopolysacchari-doses (MPS), Alkaptonuria (AKU), Hemophillia, Primary Sclerosing Cholangitis (PSC), Cystic Fibrosis, and Fragile X syndrome, as wellas a representative of EURORDIS) *Focus group: 25 patients* with Spinal Muscular Atrophy, who had recently participated in a randomized clinical cross-over trial	Not reported	Patient Think Tank (*n* = 10), and a Focus Group (*n* = 25)	Quotes from Focus Group provided and three topics identified. MAXQDA used	*Patient outcome measures:* Patients were very comfortable in talking about the practical aspects and constraints of their disease, but it was more difficult for them to answer the question of how to measure these aspects “We decided that the best results would be achieved when researchers translate the patient’s preferences in outcomes, that are formulated in the first meeting, into measurement instruments and a trial protocol which can then be evaluated again with patient representatives during the second meeting.” Subsequent presentation of a model for involving patient representatives in choosing measures during rare disease clinical trials (The POWER tool)
Gaasterland et al. [43]	Europe	To investigate *patients views on clinical trial design*	*10 educated rare disease patient representatives* [as above]	The Medical Ethics Review Committee of the Academic Medical Center has confirmed that the Medical Research Involving Human Subjects Act does not apply for this study (W17–217 # 17.249)	Interviews were conducted (*n* = 10) according to a pre-set interview guide	Grounded theory using Thematic Analysis and MAXQDA. Topics were structured in chronological order	*Involvement in trial design:* Patient organizations in which participants are involved have initiated trials, whereas other participants felt that their patient organizations were not involved in the setup of a trial early enough. These patient organizations were only approached when the trial was already recruiting, as a source of participants. Participants wanted patient organizations to be involved in the trial at an early stage *Opinions on trial design:* Participants feel they should be involved in the choice of clinically meaningful outcome measures, because patients know which outcomes are most relevant. Some thought trials that they were involved in were too short to show an effect of an intervention. Some wanted to decrease the chance of being allocated to the placebo arm of a trial. Participants do not always understand the consequences of random allocation *Trial participation:* It should be more widely known that trials are performed in hospitals in order to generate evidence about treatments. Participants need to be clear about the consequences of participation and information does not mention the practical aspects which are relevant to patients Participants make a risk–benefit analysis before taking part Participants communicate with each other and discuss treatments. Some measures are burdensome and intrusive *After the trial:* Results of a study should be clearly communicated to participants
Gengler [35]	USA	*How families* with a child with a rare disease *accessed and negotiated care* at a top 10 ranked university hospital	Strategic case selection at psuedonymised hospital. Cases reflected a variety of life-threatening childhood illnesses, and families from across geographical, educational racial, and class backgrounds	Not reported. Reported that families gave consent	Family centred ethnographic approach (Lareau 2003): Interviews and observations with *18 families*, over 18 months	Grounded theory (Charmaz 2006); and ethnographic methods (Tavory and Timmermans 2009; Prus 1987; Schwarlbe et al. 2000; Flyvberg 2001)	*Getting access:* [With Cultural Health Capital] “Todd and Savannah Marin are a case in point. When their then 6-month-old son, Jacob, was diagnosed with Tay-Sachs—a rare, fatal, genetic, degenerative neurological disorder—Savannah, a white, 32-year-old, first-time mother with a bachelor’s degree in nursing, and Todd, a white, 35-year old contractor, were devastated to learn that the only option for Jacob in their West Coast home state was palliative hospice care. Todd recalled the diagnosing physician telling them, “Go look for clinical trials—‘you go do your homework and I’ll do mine.’” But at their next visit, Savannah reported, “He hadn’t done anything. Like... he had printed out another sheet from his database...and told us, ‘Oh, looks like he has 2–4 years to live.’” Unwilling to accept this outcome, the Marin’s “did their homework” and scoured FDA databases for clinical trials.”
Kesselheim et al. [36]	USA	To explore *rare disease patients’, caregivers’, and advocates’ experiences* with their conditions and the health care system, in addition to their *perspectives on drug development*	Participants recruited via the National Organization for Rare Diseases	Not reported	Three in-person focus groups, involving rare disease patients (*n* = 9), caregivers (*n* = 8), and advocates (*n* = 9)	Grounded theory (Bradley et al. 2007), using Atlas.ti	*Concerns about clinical trial enrolment:* An important barrier for those with rare diseases, was the perceived loss of an opportunity for treatment when assigned to a control arm in a randomized trial that compared a new drug with a placebo *Narrow eligibility criteria:* limits to their access to clinical trials, due to exclusion due to co-morbidities or existing medications *Trial outcomes:* Failure to account for quality of life or outcomes driven by patients and their families *Approvals:* Participants were concerned that the development and testing of therapies should, as quickly as possible, yield effective treatments to advance their quality of life
Lopes et al. [44]	Brazil	To evaluate vulnerabilities and suggest approaches for rare disease (RD) diagnosis and treatment in Brazil based on the perceptions of those involved in the process: patients, caregivers, patient support groups, non-governmental organizations and primary and tertiary care professionals	Focus groups with 27 participants: Patients and primary care givers (*n* = 7), non-governmental associations and organizations (*n* = 9), primary care professionals (*n* = 4), physician specialists (*n* = 7)	Ethics Committee for Review of Research Projects (Comitê de Ética para Análise de Projetos de Pesquisa—CAPPesq) of Hospital das Clínicas of the FMUSP (268.417)	Non-random Sampling (*n* = 7). After the study objectives were presented, all the participants signed an informed consent form Researchers involved in the study served as moderators and reporters in each group to ensure procedural homogeneity A script containing guidelines was presented to each group before the session was initiated	Reports were recorded and transcribed in full and served as a basis for the thematic and categorical content analysis (Bomfim 2009, Oliveira 2008). Thematic units were coded and processed with NVivo 10 software, which allowed us to map their distribution among the different study groups (Bardin 2007, QRS 2013). For better reliability, data triangulation was used to achieve the highest degree of convergence among the researchers’ perceptions	*Diagnosis:* The patient’s journey does not end with the diagnosis of the disease; another significant obstacle faces health personnel and relatives after diagnosis: the challenge of searching for adequate treatment, which, at least in Brazil, is far from straightforward. The Brazilian public health system has been unable to meet the needs of RD patients who, due to the multiplicity of these diseases and their rarity, are frequently unable to organize a strong and representative support group *Treatment:* Regarding medications for RDs or ‘‘orphan drugs’’, there is a lack of incentive for the national pharmaceutical industry to conduct research and testing related to the manufacture of these medications. Consequently, these drugs must be imported, which leads to greater public costs Additional needs of RD patients could be met if appropriate technologies were directed toward research aimed at developing new, more accessible and affordable therapies
Li et al. [45]	China	To assess the unmet needs of rare disease patient organizations in China, and identify their unmet needs, providing essential information for the government and legislators	28 participants, representing 28 patient organizations for rare diseases	Institutional Ethics Committee of the Guangzhou Medical University, China. All interviewees signed the informed consent and agreed to participate in this study voluntarily	Participants (*n* = 28) were recruited through online advertisements or personal references. Interviews were conducted by phone and each participant asked 18 questions	Common themes from these transcripts were analysed in this study	*Research:* Patient organizations have not been able to establish registries or sponsor research due to lack of financial support. None of the participants in the study had been involved in research
Menon et al. [38]	Canada	The aims of this study were (1) to *explore opportunities for patient involvement in reducing decision uncertainties throughout the lifecycle of orphan and ultra orphan drugs* from the perspectives of patients within the Canadian rare disease community; and (2) to develop a policy framework for patient input that maximizes the impact of their involvement on decision uncertainties around orphan and ultra-orphan drugs	Two one-day conferences and four workshops involving patients and/or families from rare disease communities in Canada were held to discuss issues around orphan and ultra-orphan drug development, access, and coverage, and identify opportunities for patient input to reduce related decision uncertainties	The project was approved by the University of Alberta Health Research Ethics Board	*Conference #1:* Presentations were followed by small group sessions with participants from patient communities (*n* = 60), who were asked to discuss the goals of an ‘ideal’ process for managing the development and introduction of new therapies *Conference #2:* Participants included patients and families, clinical specialists in rare diseases, and representatives from industry, Health Canada (federal regulatory body), and the provincial governments (payers). Of the patient participants (*n* = 69), most had attended the first conference *First 2 workshops:* Patients an family who had attended conference # 2 (*n* = 30), to explore roles specifically for patients, families, and patient organizations in reducing decision uncertainties throughout the technology lifecycle *Second 2 workshops:* All patients and families attending the fora were invited to participate in the workshops (Toronto, *n* = 23; Vancouver, *n* = 18). None had been involved in the previous sessions	Transcripts from all four workshops were analysed qualitatively using a general inductive approach (Ritchie et al. 1994). 2 researchers first read through all of the transcripts and developed initial coding categories, which represented potential themes. Chunks of text were then assigned to one or more of these categories	*Patient registries*: Are seen as an important tool to monitor rare diseases and their treatment. Registries would be an efficient way of getting large enough numbers of patients for meaningful statistics, the lack of which is often an obstacle to getting an orphan drug funded *Reimbursement process:* In Canada, coverage for orphan and ultra-orphan drugs is viewed by patients and families as one of the main issues affecting the rare disease community Other complaints included a lack of an individual patient’s voice during reimbursement review meetings, the absence of a truly fair appeal process and opacity in the decision parameters that seem to be used by committees *Value Definition:* As experts in their own disease, patients and families felt that they were best able to judge improvements in or worsening health. The choice of outcome measures in trials often reflects what is easy to measure, rather than what is of value to patients and families. As a result, the trials fail to capture the true value of a therapy *Clinical Trials:* ‘‘Because we’re talking about all the problems that happen after clinical trials are designed by people who know the science and the industry, but don’t know the disease and that’s the problem. We’re dealing with the problems because we’re not included before the trial begins.’’ Patients and families were frustrated by the lack of opportunities for patient input into the design of trials, given that they are increasingly being asked to help identify potential patients or participate in the trials themselves *Benefit –Harm Assessment:* Throughout the technology lifecycle, decisions around what constitutes acceptable harm in order to achieve a certain magnitude of benefit are often made with little input from patients. The existing paternalistic approach needs to be replaced with one that recognizes patients and families as empowered equal partners in such decisions *Early Studies and Drug Discovery (R&D):* For many patients and families living with rare diseases, there are no treatments beyond supportive care. Therefore, the need for research that could lead to the development of new therapies was a dominant theme shared by all 4 workshops. Given that resources available for research are limited, patients and families felt that they should have a role in setting research funding priorities to ensure that decisions reflect a comprehensive understanding of the potential implications of different proposals *Managed Access:* comprises a conditional funding option, which makes therapies available to eligible patients on the basis of an agreement. The terms of which may include collection of certain types of data or specific clinical or financial outcomes that must be achieved in order to receive continued funding
Peay et al. [37]	USA	Although our initial aim was to *explore the experience of parents* and clinician investigators involved in a clinical trial for a rare disorder, we were also able to *explore participation in a trial that came to an abrupt, unexpected end*	*6 fathers and 6 mothers* of 11 boys with Duchenne and Becker Muscular Dystrophy (*including 1 mother–father pair*) and 9 clinical investigators. All had participated in the phase II clinical trial. All participated at US study sites. Recruited through advocacy organisations and snowballing	Not reported	Semi structured telephone interviews with clinical investigators and parents of sons with DBMD who participated in the phase IIa or IIb ataluren clinical trial in the United States. The topics explored during the interviews – experiences in the trial, hopes, and expectations; perceptions of benefit; and relationships among stakeholders – were informed by the literature and clinical and anecdotal experience. Because these sources suggested that expectations and hopes for a clinical trial may differ, we asked participants to describe both their hopes and expectations	Using NVivo 8 QSR, the responses were analyzed by two independent investigators (T.F. and E.B.) to ensure coding consistency and high intercoder reliability Discrepancies in the coding were discussed until reconciliation was achieved. All analyses were based on consensus codes. We conducted thematic analysis within and between the parent group and the clinician investigator group. Major themes that arose from the analysis and illustrative quotes are presented	*Expectations and hopes for the clinical trial:* Parents reported and demonstrated being well informed about the trial. All reported expecting some direct benefit of the drug, usually described as slowing or stabilizing progression of the disorder *Motivations and decision making:* Parents’ primary motivation for enrolling was the potential for benefit. Less than 1/2 of the parents mentioned altruism. Most of the parents reported an easy decision or ‘non-decision’ to join the clinical trial *Pressures of a progressive disorder:* Parents spoke about the pressures of a progressive, fatal disorder, and how these pressures played a role in decisions about and expectations of clinical trials *Perceptions of benefits:* The parents delineated direct and indirect benefits of trial participation. All reported some degree of direct benefit for their boys, ranging from obvious improvements to subtle changes. These benefits included improved strength, endurance, and cognitive performance. A few parents described being unsure about whether there was benefit until they noted declines following the sudden end of access to the drug *Reactions to trial ending:* Parents reported anger, shock, and distress when the trial was stopped. Several parents noted the need to better prepare participants for the possibility of a trial ending abruptly
Tingley et al. [39]	Canada	To integrate perspectives from published literature and key rare disease stakeholders *to better understand the perceived challenges and proposed methodological approaches to research on clinical interventions for rare diseases*	Recruitment invitations were distributed by email to physician members of the Garrod Association (a professional association whose members are involved in caring for patients with inherited metabolic diseases), to policy advisors by a member of their professional network (using publicly available contact information), and to patients/ caregivers attending the Canadian MPS Society’s 2017 Annual Family Meeting Individuals interested in participating were instructed to contact a member of the research team (KT), and eligible respondents were asked to provide signed, informed consent to participate in the study	Approved by the Ottawa Health Science Network Research Ethics Board and the Children’s Hospital of Eastern Ontario Research Ethics Board (physicians and policy advisors), and the University of Ottawa Health Sciences and Sciences Research Ethics Board (patients/caregivers)	Literature review, plus: Focus group interviews were conducted by telephone with the physicians (*n* = 6) and policy advisors (*n* = 3), and in-person with the patients/caregivers (*n* = 4) in conjunction with the Canadian MPS Society’s 2017 Annual Family Meeting held in Montreal, QC, Canada	Each focus group transcript was analyzed using a qualitative descriptive approach that is aimed at “obtaining straight and largely unadorned (i.e., minimally theorized or otherwise transformed or spun) answers to questions of special relevance to practitioners and policy makers” (Sandalowski 2000). Four members of the study team (KT, BP, DC, IG) met to identify the key concepts and themes that were present in the focus group data. These concepts/ themes were organized into a coding system that was applied by one study team member (KT) using NVivo 10 Software (QSR International Pty Ltd.) and reviewed by a second member (BP) for credibility and trustworthiness (Shenton 2004)	*Explanatory evidence generation:* Participants in our focus groups highlighted the limited feasibility of conventional RCTs because of small sample sizes, but there was little emphasis on specific strategies that might be used to overcome this challenge There can be a lack of patient/ family/ clinician acceptance of the possibility of being randomized to a control group, particularly for placebo controlled studies of treatments for rare diseases where few treatment alternatives exist. Therefore, study designs that make participation more appealing by maximizing time spent on- or guaranteeing provision of- the active treatment have been suggested Studies designed to evaluate the efficacy of an intervention typically limit enrolment to a very homogenous group of participants, which strengthens the robustness of the causal interpretation of the findings, but at the expense of a reduction in the external validity or generalizability of study results *Comparative effectiveness/ pragmatic evidence generation:* clinical heterogeneity is often not accounted for in conventional RCTs, and has raised concern among stakeholders about the applicability of study results to patients with clinical manifestations different from those included in RCTs Several focus group participants have advocated for study designs that may compromise internal validity to some extent, by shifting away from the explanatory RCT, in order to address real-world effectiveness Participants questioned the suitability of explanatory RCTs for establishing effectiveness of clinical interventions for rare diseases, but little of the discussion focused on specific solutions to overcome this challenge *Patient-oriented evidence generation:* One of the main criticisms, by focus group participants, is of highly internally valid, explanatory study designs is their tendency to rely on short term, and often surrogate, outcomes that are not necessarily clinically meaningful. Many outcome measures, including patient-oriented outcome measures, have not been validated or standardized for the population of interest, leading to questions about the applicability of study results Some participants expressed concern about balancing subjective outcomes (e.g., patient-reported quality of life) with more objective outcomes (e.g., biomarkers of disease progression) because of possible placebo effects with patient-reported outcomes
Tingley et al. [40]	Canada	*To understand why and how patients and families* with rare metabolic diseases, specialist metabolic physicians, and health policy advisors *choose whether to participate in studies and how they use and value research*	We distributed recruitment invitations by email to physician members of the Garrod Association (a professional organization whose members are involved in caring for children with IMD), to policy advisors by a member of their professional network using publicly available contact information, and to patients/caregivers attending the Canadian MPS’s Society’s 2017 annual Family Meeting Individuals interested in participating in the focus groups contacted the lead author (KT) for more information and to confirm eligibility. Eligible respondents were asked to provide signed informed consent to participate in the study	Approved by the Ottawa Health Science Network Research Ethics Board, the Children’s Hospital of Eastern Ontario Research Ethics Board, and the University of Ottawa Health Sciences and Sciences Research Ethics Board. Informed consent was received from all participants in this study	Focus group were held separately for each stakeholder group; the patient and family focus group was conducted in person in conjunction with the Canadian MPS Society’s 2017 Annual Family Meeting, We reasoned an in-person focus group may be important for patients and families We developed a semi-structured interview guide for each focus group that included questions related to the generation and evaluation of evidence for clinical interventions for rare diseases. Topics included: general perspectives on rare disease research, reasons for participating in research activities, outcomes used in clinical studies, and challenges in establishing treatment efficacy and effectiveness One team member (KT) conducted all three focus group interviews, with at least 1 additional team member attending as an observer We completed three focus group interviews with a total of 13 participants (physicians *n* = 6; policy advisors *n* = 3; patients/caregivers *n* = 4). Focus group interviews lasted between 45 and 75 min	Each interview was audio-recorded with participants’ consent and transcribed for data analysis. The transcripts were analyzed sequentially using thematic analysis (Braun et al. 2008), which involved generating a set of initial codes based on interesting features of the data and then organizing those codes into key themes related to the research topic. To do this, a series of research team meetings were held to review the transcripts and inductively identify emerging concepts from each interview. Key concepts that were identified in the focus group data were organized into a coding system that was applied by one member of the study team (KT) using NVivo 10 software (QSR International Pty Ltd.) across the entire data set. Coded transcripts were reviewed and verified by a second team member (BP) to confirm all codes had been applied appropriately and that no themes had been overlooked. We used several strategies to ensure credibility and trustworthiness of our data (Shenton et al. 2004), including: debriefing sessions after each focus group to identify key perspectives, multiple	*Making choices about participating in research:* Patients and caregivers did not explicitly mention choosing to participate based on gaining access to treatment. Participants viewed involvement in research activities as a form of advancing science and an act of altruism of potential benefit to the next generation of individuals affected by the disease, understanding that they or their family members may be unlikely to personally benefit Patients and/or caregivers also described approaching research as an opportunity to share their own experiences and in turn to benefit from research findings that describe a broad range of experiences of other patients and families, to help inform decision-making Not all patients and caregivers found it easy to make the decision to participate in a research study or to try a new therapy. Participants described being fearful of making the wrong decision in choosing whether to participate Patients and their caregivers also reported difficulty with weighing the risks versus benefits of trying a new therapy and spoke about uncertainty about whether it would be “worth it” Despite the difficulties and uncertainty in making decisions to participate in clinical research or to try an experimental treatment, one caregiver highlighted the importance of being persistent and continuing to ask questions and do research Patients or caregivers expressed a desire for research to be more accessible and noted that sharing findings from research in which they’ve directly participated may help encourage further research engagement *Perspectives on the value of research:* one concern that was raised in all three groups with respect to the quality of research was the difficulty of conducting high quality rare disease studies due to limited resources. In addition, participants across groups were concerned about potential bias in studies that are solely funded by pharmaceutical companies
Young et al. [41]	Canada	*To explore ways in which* Canadian *patients* with rare diseases and their families would *like to be involved in the lifecycle of therapies and identify their priorities for involvement*	Patients with rare diseases and their families were recruited to participate in two deliberative sessions, during which concepts related to decision-making uncertainty and the technology lifecycle were introduced before eliciting input around ways in which they could be involved. This was followed by a webinar, which was used to further identify opportunities for involvement	Approval from the University of Alberta Research Ethics Board, project name ‘Patient Preferences around Therapies for Rare Disease’, no. MS1_Pro00029603, 3 September 2014. All procedures performed in studies involving human participants were in accordance with the ethical standards of the University of Alberta Research Ethics Board and with the 1964 Helsinki declaration and its later amendments or comparable ethical standards. Informed consent was obtained from all individual participants included in the study	Pragmatic qualitative research methods were used in which the methods selected for data collection and analysis were those most likely to provide insights into the research question, without adherence to any specific research approach (Savin-Baden 2013) Patients and families (participants) were recruited through two national events of the Canadian Organization for Rare Disorders (CORD), Canada’s national network for organizations representing those with rare disorders *Deliberative session 1:* Total participants *n* = 118, including health professionals, industry and government (patients, and their family members, and/or patient representatives (*n* = 46) *Deliberative session 2:* Participants are all patients, and their family members, and/or patient representatives (*n* = 14) *Webinar:* Participants are all patients, and their family members, and/or patient representatives (*n* = 8)	One researcher (AY) thematically analyzed the audio recordings and field notes from the sessions using eclectic coding (Saldana 2012). A second researcher (TS) analyzed an overlap of 25% of the recordings and field notes using the same methods to ensure validity of coding. Descriptive and process coding were used to identify the topic (e.g. coverage decision making) and the activity (e.g. providing input), respectively. This yielded a list of activities that patients and families felt they could be involved in However, additional information on these activities was obtained using values coding (reflecting perspectives, values, attitudes or beliefs about the specific type of engagement), evaluation coding (assigning judgments about the merit, worth, or significance of programs or policy), and emotion coding (labeling the emotions recalled and/or experienced by the patients/families, or inferred by the researcher about the patients/families). The activities were further grouped into goals that patients and families hoped to achieve by participating in those activities identified	*Patients or family members should provide input into clinical trial design, including identifying and selecting meaningful outcome measures:* Some issues that occur later on in the lifecycle could be avoided by having patients, who are experts in their diseases, involved in the design of the trial to ensure that relevant data is collected It is frustrating that there appears to be no consideration of the endpoints that will be meaningful to reimbursement decision-makers earlier on It is not feasible to bring every patient or family member to the table to select meaningful outcome measures, but it is still necessary to have some input These endpoints need to be well-defined *Patients should be involved in interpreting the meaningfulness of the data collected:* The value that patients place on the benefits that they experience in a trial will be different than the value others (e.g., payers; society; etc.) will place on those benefits It’s important that their input be used to capture the meaningfulness of the outcomes collected in a trial *Patients should participate in clinical trials:* Orphan drugs often do not have a strong evidence base but patients are willing to participate in trials regardless *Patients should submit patient-reported outcome measures (PROMs) during clinical trials:* In many clinical trials, the clinical outcomes that data were collected on did not capture the positive benefits that they experienced on a new drug. This is frustrating, as the data that is then considered by reimbursement decision-makers is incomplete Having the ability to report on these benefits provides important data for decision-making *Patients should adhere to the treatment protocol:* This has been an issue in the past where patients were less compliant with more burdensome treatments, negatively affecting their outcomes

Papers included patient and public involvement and engagement (PPIE) activities with patient representatives from umbrella patient organisations, as well as qualitative research with individual patients or caregivers from single disease organisations or attending clinics; but sometimes the boundaries between PPIE and research were blurred (e.g. stakeholder activities without research ethics approval presented as ‘data’). Most papers included identifiable approaches to qualitative data collection [36, 37]; however, counter to reporting guidance for qualitative research [25], only one paper [35] included an overarching methodological framework, with some citing reporting guidance, rather than qualitative methodological literature, as informing their research design [43]. Few papers provided details of how the authors conducted their qualitative analyses, although some reported findings statistically [33]. Some papers were more akin to reports of patient and public involvement/engagement (PPIE) workshops, or stakeholder events with mixed patient and clinician populations [44]. Few papers detailed the relationship between the author and participants, with limited reflexivity about their role in the construction of the research findings [21, 25]; thus making it difficult to discern how patients and other members of the public contributed to the generation of substantive knowledge about patient engagement in orphan drug development.

The 4 substantive headings, below, were those most used by the authors of the papers under review (as sub-headings) in their interpretations of the stakeholder perspectives in the primary papers, and typically follow the chronological processes of clinical trial and drug development [46, 47].

### Trial design

Earlier research identified that patients with rare diseases want to see the adoption of a faster approval processes of new therapeutic agents that would produce effective treatments and improve their quality of life [36]. More recently, attention has turned to how patients, as experts in their own disease, can be active agents in the development of trial protocols, rather than merely as trial recipients [38, 41, 43]:

‘‘Because we’re talking about all the problems that happen after clinical trials are designed by people who know the science and the industry, but don’t know the disease and that’s the problem. We’re dealing with the problems because we’re not included before the trial begins.’’ (Participant, Menon et al 2015: 108).

Patient representatives reported negative perceptions of conventional randomised control trial designs and placebo controlled studies [39]. Instead, they suggested that their involvement in the trial design and protocol development could mitigate burdensome treatment regimens [41], and widen the parameters of enrolment to ensure that findings had increased external validity and thus applicability to a wider patient population [39].

Several papers suggested that patients and caregivers are not prepared to accept the outcome measures and clinical endpoints which trial designers’ currently offer [35, 39], and which fail to adequately account for quality of life [36]. The ‘true value’ of a therapy can therefore be lost because current outcome measures focus on what is easy to measure [38], or use standardised measures, rather than focusing on outcomes of interest to specific populations [39]. Authors also described the difficulty in eliciting patient outcomes and the need for new models of outcome development [42]:

“We [authors] decided that the best results would be achieved when researchers translate the patient’s preferences in outcomes, which are formulated in the first meeting, into measurement instruments and a trial protocol which can then be evaluated again with patient representatives during the second meeting.” (Gaasterland et al 2018: 1290)

The selection of clinical endpoints that were not considered as meaningful to reimbursement decision-makers was also a cause of frustration [41], and patient representatives identified that they should have a greater role in both research and reimbursement funding priorities [38].

### Trial access

Patient representatives expressed disappointment when inclusion criteria, such as the phase of a disease, co-morbidities and existing medication regimens inhibit enrolment [33, 36]. Clinicians were viewed as gatekeepers, who can limit the enrolment of patients from minority backgrounds, due to beliefs that they will be unable to fulfil the research objectives or be non-compliant [33]. Lack of sponsorship can ensure that patients require sufficient ‘cultural health capital’, in order to push for access to a clinical trial [35]:

“Todd recalled the diagnosing physician telling them, “Go look for clinical trials—‘you go do your homework and I’ll do mine.’” But at their next visit, Savannah reported, “He hadn’t done anything. Like . . . he had printed out another sheet from his database . . . and told us, ‘Oh, looks like he has 2–4 years to live.’” Unwilling to accept this outcome, the Marins “did their homework” and scoured FDA databases for clinical trials.” (Gengler 2014: 346)

Several papers described the burden on patients of travelling to and from study appointments [34], which was magnified when parents of disabled children were required to stay in unfamiliar places without support networks [33]. Proposed solutions included financial remuneration, but also flexibility [34]:

“I might consider doing it depending on the leniency of when I could come in or what hours can I come into the office. I would be more drawn to a study that brought me in fewer times a week” (Participant, Carroll et al 2012:7)

A key motivator for trial participation was disease progression and associated high expectations for the benefit of trial medicines [37], even when patients themselves were unlikely to derive individual benefit [40]. In countries, yet to establish registries and normalise trial delivery, wider financial incentives were called for [44, 45].

### Trial participation

While patient registries were seen as an important tool for monitoring rare diseases, and a means to recruit sufficient participants to a trial [38, 45], there were also concerns that they are only ‘suitable for highly motivated or informed volunteers who are specifically interested in research’ [33].

Despite the lack of a strong evidence base, rare disease patients are often willing to participate in trials of new drugs, even when they perceive that improvement may be minimal [36, 41]:

“There isn’t enough available for you to be able to prioritize, and so therefore you will grasp at anything that is acceptable to you. It doesn’t matter if it’s going to perhaps improve by 1% or by 50% or will get to the cure level of 100%. You will take it.” (Advocate, Kessselheim et al 2014: 78)

Throughout the drug lifecycle, decisions around what constitutes acceptable harm in order to achieve a certain magnitude of benefit are traditionally made with minimal input from patients, suggesting that there is significant scope for patients and families to be engaged as equal partners in such decisions [38]. Patients have suggested that they are concerned about the side effects of experimental medicines, and the consequences of stopping current drug regimens [34]; while others have expressed fears about making the ‘wrong’ decision, in agreeing to participate in a trial or not [41]. This can be compounded when patients are assigned to a control arm in a trial, and when the perception of any ‘potential gain’ is diminished [36]. Other patients have suggested that they have only been able to weigh up risks and harms retrospectively, that is after a trial, and specifically when any perceived improvement is subsequently lost [33].

Significant attention has also been given to the potential role of patient representatives in the design of patient-facing information. Patients and their caregivers often perceive that there is an over-abundance of information, which is fragmented, difficult to obtain, or difficult to understand [33] and which, paradoxically, can obfuscate that trials are performed in hospital with the objective of generating evidence about the effectiveness of treatments [43]. In the one example, where a trial was terminated before completion, parents reported feeling powerless, as their sense of hope receded [38]:

When he called up and said stop taking the medicine, I felt that conversation was worse than the diagnosis phone call when they told me he had muscular dystrophy... hope goes a long way, and to take that from a family is just pretty devastating ... The shattering part was because it was his cure. (Father 107, Peay et al 2014: 82)

In this example, parents were not prepared for a common trial outcome (no effect on the primary trial endpoint), which was compounded by the desperation wrought by the lack of available treatment (often typical for the rare disease community), and exacerbated by perceptions that the trial drug was of benefit for their children (in the absence of alternatives). It is suggested that patient facing information needs to better prepare participants for the possibility of a trial ending abruptly [38]; and patients have suggested that peer support and the use of social media could be used provide patient-to-patient information and support [33, 40, 42]

### Dissemination

Two recent papers acknowledge that patient engagement activities typically end when their participation in a clinical trial finishes [40, 43]. Participants suggest that the results of a study need to be communicated to patients, both more often and more clearly [40, 41]:

“…I think that’s a really important piece to keep people motivated to participate in these things is to at least have some sort of follow through that allows us to see if what we shared made any kind of a difference. So that would be one thing that I’d like to see…”(Patient/caregiver 4, Tingley et al 2021: 6).

This would enable patients to evaluate their contribution to the research, as well as potentially encourage future participation in research [40].

## Discussion

By formally appraising qualitative studies about how members of the public engage with orphan drug development, and employing the framework developed by Brown and Bahri [12], we identified a lack of *depth* in existing studies, due to the lack of understanding and rigorous application of methodologically informed qualitative research [48, 49]. Limitations include: the conflation of patient and public involvement and engagement (PPIE) and qualitative research, and a lack of detail pertaining to recognised qualitative analysis techniques (e.g. thematic or narrative analysis), and limited reflexivity about the authors relationship to those being researched [21, 26]. We also identified the adoption of a range of more *quantitative* techniques within papers purporting to have used qualitative methods. These include: interviews no longer than 10 min in length [34], the use of closed questions [44], and statistical analyses [33], which inhibited the development of richer understandings of patients’ unmet needs.

Recent research undertaken by EURODIS has identified that a barrier to sustaining meaningful patient engagement in rare disease research is a lack of knowledge on how to apply methodologies to capture and use patient insights [50]. More specifically, du Plessis et al. [51] have identified a lack of understanding of, and respect for, qualitative research methods that often form the foundation of patient-centric research in drug development. This lack of methodological knowledge matters, when engagement is becoming the new standard for patient-facing clinical trials and the development of new medicines to address patient needs [52].

Our analysis identified that patients and caregivers need to be involved in study design (including protocol development and the selection of measures that capture a range of outcomes, e.g. quality of life, beyond clinical endpoints) to optimise recruitment and demonstrate effectiveness. Patients and caregiver input is also necessary to ensure that trial providers enable access to the trial, develop patient facing materials that facilitate meaningful decision making, and disseminate results to patients to acknowledge their contribution and foster future collaboration. That these findings align with critical junctures for patient engagement identified in existent roadmaps for the drug development pipeline, developed by rare disease patient organisations is unsurprising [50, 52].

Earlier research about patient organisations generally, and those for rare diseases specifically, identified their imperative to engage with, and sometimes dependence upon, both the agenda and financial sponsorship of pharmaceutical organisations [53–55]. However, the roadmap developers are clear that these plans remain aspirational, rather than realised; with examples in the wider literature of sub-optimal patient engagement, in terms of lack of opportunities for genuine involvement in decision making [56], and acknowledgment that the patients who get to have their say in engagement activities are not representative of wider patients and publics [57, 58].

Accessing the perspectives of those who do not wish to participate in research is a so called a ‘wicked problem’ [59]. Two of the authors (JF and CP) are now undertaking a qualitative study of patients with one rare disease who have participated in, declined to participate in, or (been) withdrawn from, trials of new medicines and observational studies. We acknowledge, that in our focused study, we are only able to capture the perspective of those have agreed to be interviewed. However, to date we have interviewed patients with experience of participating in clinical trials and patients organisations, as well as those who have refused to participate in a trial, who have been declined the opportunity to participate in a trial because they have not met the inclusion criteria, as well as those who have withdrawn from a trial, and those who are not sure if they have been invited to, or participated in, research.

We suggest that more creative social science and participatory approaches might better enable those who are risk exclusion by traditional health services research methods from participating. See for example the recent initiatives from UK NIHR INCLUDE frameworks for ethnicity, and for people who lack capacity to consent [60].

Through using Brown and Bahri’s framework [12], we detected a lack of *breadth* in existing qualitative studies, due to the lack of diversity in the patients that have been engaged both socio-economically and geographically, and a narrow focus on the established ways of augmenting, rather than redefining, established clinical trial research [61]. For example, little or no attention in trial design is currently given to the perspectives of patients who do not want to be involved in research. More recently, Galasso and Geiger have suggested that innovation in precision and genomic medicine development risks exacerbating health care inequalities by benefitting people who are already advantaged [62]. They propose a more participatory medicine that would allow a wider public to voice their dissatisfaction, rather than being not- yet-reached or still-not-heard as at present; which might enable a more inclusive and democratic form of innovation [62].

In terms of *texture*, the third dimension of Brown and Bahri’s framework [12], we identified a lack of attention to both the processes of research and processes of engagement. This is important because, as Brown and Bahri suggest, understanding the processes of reactions to a new indication, the mediating factors of experience (e.g. trust, time, and resources), and any unintended consequences or barriers, are crucial to the design of effective measures and analyses of outcomes [12]. For example, although COVID has brought new opportunities for the use of remote and digital technologies for trial designs in rare diseases [63], attention must also be paid to which patients risk being further disenfranchised by the introduction of these methods.

Qualitative research is rooted in the philosophy of interpretivism, and may employ a range of methodological approaches that enable the interrogation of people’s views and perspectives, as well as any taken-for granted assumptions that may inform or provoke them [64]. We identified an absence of methodological frameworks to inform the research design and instead authors employed generic or off-the-shelf methods (such as ‘interviews’) [65], with some authors taking the patient and public perspectives at face value rather than providing an analysis [42]. Only one paper provided a methodological framework to inform data collection and analysis [35], and none engaged with the principle of reflexivity. Thus, absence in the detail and nature of the relationship between the researcher and researched ensured that any power dynamics involved typically remained obscured [66].

Considering the motives for the research were often to explore patient and caregiver perspectives, it is noteworthy that none of the studies were co-designed with patients or caregivers, nor patient-led. None of the papers reported critical reflection on the mechanisms of engagement activities [67–69], and instead presented case-studies as a sufficient reporting mechanism [56]. We contend that the texture of patient and public engagement in orphan drug development would be considerably improved if those researching their experiences and perspectives employed qualitative methodology, rather than ad-hoc methods, and ensured that the processes and outcomes of engagement are fit for purpose (e.g. inclusive and impactful).

**Fig. 4:**
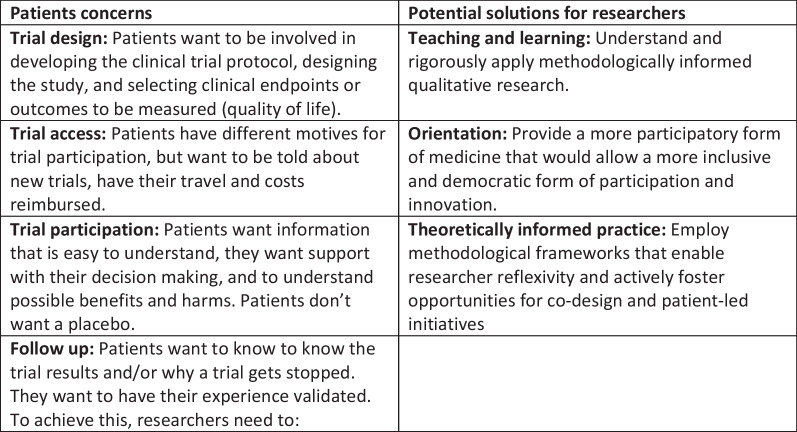
Key concerns and suggestions for how to address them

In sum, we were able to identify an uneven landscape in the topography of public engagement that is indicative of both the complexity of researching rare diseases in different health care systems and using trial designs, but also the importance of structural inequalities. Data are ‘missing’ from our analysis, because of the methodological weaknesses in the papers or because some kinds of patient are absent from activities conducted with established patient organisations. However, our qualitative synthesis identified several key concerns of patients and has enabled us to suggest how future researchers may address the gaps identified (Fig. 4).

### Strength and limitations

A limitation of this research is that a wider literature search may have enabled us to understand more fully the landscape of how patients and other members of the public are currently engaged in orphan drug development. Furthermore, that the findings of our analysis were not robust enough for us to undertake a meta-ethnography is not indicative of failure. Having employed relevant search filters, and then focused upon the four highly discriminating methodological MeSH terms, we were able to evaluate any methodological weaknesses, using validated tools, and discern the substantive contributions in arguably the most relevant examples in the current qualitative literature.

A strength of this research is that we engaged with stakeholders throughout the research process. This included regular input into the design and content of the research from the Patient Advisory Group and steering meetings, as well as in presentations to industry partners and with colleagues working in clinical practice. This has enabled us to make some pragmatic recommendations about both the conduct of qualitative research and public engagement.

## Conclusion

Conducting a qualitative synthesis of how patients and other members of the public engage with the orphan drug development, informed by Brown and Bahri’s framework, enabled us to identify the explicit need for methodological rigour in research with patients with rare diseases. This includes the need for appropriate and innovative use of qualitative methods and distinct PPI activities (rather than their conflation) and more strenuous efforts to capture the perspectives of under-served -researched or seldom-listened to communities with experience of rare diseases. This latter focus will require more creative recruitment and wider adoption of post-colonial practises, and a re-alignment of the research agenda (to encourage the use of co-design to enable patients to set the agenda, rather than respond to what they are being offered).

## Supplementary Information

**Additional file 1. Supplementary material: **Results of highly discriminating search (full texts retrieved)

## Data Availability

The datasets used and/or analysed during the current study are available from the corresponding author on reasonable request.
