# Supplementary material for: How do patients and other members of the public engage with the orphan drug development? A narrative qualitative synthesis
Source: Orphanet J Rare Dis. 2023 Apr 17;18:84. doi: 10.1186/s13023-023-02682-w (PMC10108537; doi:10.1186/s13023-023-02682-w)
Supplement: Supplementary file 1 — Additional file 1. Supplementary material: Results of highly discriminating search (full texts retrieved) [file 13023_2023_2682_MOESM1_ESM.docx]

**Supplementary material: Results of highly discriminating search (full texts retrieved)**

Aksu, N. B. and B. Eren (2019). "Rare Diseases and Orphan Drugs." *Journal of Allied Pharmaceutical Sciences.* 1 (2); 1-3.

Albright, K., et al. (2016). "Seeking and sharing: why the pulmonary fibrosis community engages the web 2.0 environment." *BMC Pulmonary Medicine* 16: 4.

Austin, C. P., et al. (2018). "Future of Rare Diseases Research 2017-2027: An IRDiRC Perspective." *Clinical and Translational Science* 11(1): 21-27.

Bendixen, R. M., et al. (2016). "Engaging Participants in Rare Disease Research: A Qualitative Study of Duchenne Muscular Dystrophy." *Clinical Therapeutics* 38(6): 1474-1484.e1472.

Bevan, J. L., et al. (2003). "Informed lay preferences for delivery of racially varied pharmacogenomics." *Genetics in Medicine: official journal of the American College of Medical Genetics* 5(5): 393-399.

Brenna, E., et al. (2020). "The implementation of health technology assessment principles in public decisions concerning orphan drugs." *European Journal of Clinical Pharmacology* 76:755–764.

Bronstein, M. G. and E. D. Kakkis (2016). "Patients as key partners in rare disease drug development." Nature reviews. *Drug Discovery* 15(11): 731-732.

Carroll, R., et al. (2012). "Motivations of patients with pulmonary arterial hypertension to participate in randomized clinical trials." *Clinical Trials* 9(3): 348-357.

Contesse, M. G., et al. (2019). "The Case for the Use of Patient and Caregiver Perception of Change Assessments in Rare Disease Clinical Trials: A Methodologic Overview." *Advances in Therapy* 36(5): 997-1010.

Crossnohere, N. L., et al. (2020). "The evolution of patient-focused drug development and Duchenne muscular dystrophy." *Expert Review of Pharmacoeconomics & Outcomes Research* 20(1): 57-68.

de Freitas, C., et al. (2021). "Public and patient involvement in health data governance (DATAGov): protocol of a people-centred, mixed-methods study on data use and sharing for rare diseases care and research." *BMJ Open* 11(3): e044289.

de Vries, S. T., et al. (2017). "Factors Influencing the Use of a Mobile App for Reporting Adverse Drug Reactions and Receiving Safety Information: A Qualitative Study." *Drug Safety* 40(5): 443-455.

Dockser Marcus, A. (2009). "To make progress in rare cancers, patients must lead the way." *Journal of clinical oncology : official journal of the American Society of Clinical Oncology* 27(16): 2575-2577.

Douglas, C. M. W., et al. (2015). "Why orphan drug coverage reimbursement decision-making needs patient and public involvement." *Health Policy* 119(5): 588-596.

Fischer, J. and H. M. Van de Bovenkamp (2019). "The challenge of democratic patient representation: Understanding the representation work of patient organizations through methodological triangulation." *Health policy* 123(1): 109-114.

Forsythe, L. P., et al. (2014). "A systematic review of approaches for engaging patients for research on rare diseases." *Journal of General Internal Medicine* 29 Suppl 3: S788-800.

Gaasterland, C. M. W., et al. (2018). "The POWER-tool: Recommendations for involving patient representatives in choosing relevant outcome measures during rare disease clinical trial design." *Health policy* 122(12): 1287-1294.

Gaasterland, C. M. W., et al. (2019). "The patient's view on rare disease trial design - a qualitative study." *Orphanet Journal of Rare Diseases* 14(1): 31.

Gargiulo, M., et al. (2013). "Attitudes and expectations of patients with neuromuscular diseases about their participation in a clinical trial." *Revue Neurologique* 169(8-9): 670-676.

Gayet-Ageron, A., et al. (2020). "Study design factors influencing patients' willingness to participate in clinical research: a randomised vignette-based study." *BMC Medical Research Methodology* 20(1): 93.

Gengler, A. M. (2014). ""I want you to save my kid!": Illness management strategies, access, and inequality at an elite university research hospital." *Journal of Health and Social Behavior* 55(3): 342-359.

Groot, B., et al. (2021). "Adolescents' experiences with patient engagement in respiratory medicine." *Pediatric Pulmonology* 56(1): 211-216.

Hoekstra, F., et al. (2020). "A review of reviews on principles, strategies, outcomes and impacts of research partnerships approaches: a first step in synthesising the research partnership: approaches: a first step in synthesising the research partnership literature. *Health Research Policy and Systems.* 18:51

Ienca, M. and E. Vayena (2020). ""Hunting Down My Son's Killer": New Roles of Patients in Treatment Discovery and Ethical Uncertainty." *Journal of Bioethical Inquiry* 17(1): 37-47.

Iriart, J. A. B., et al. (2019). "From the search for diagnosis to treatment uncertainties: challenges of care for rare genetic diseases in Brazil." *Ciencia & Saude Coletiva*. 24(10): 3637-3650.

Janssens, R., et al. (2018). "Patient involvement in the lifecycle of medicines according to Belgian stakeholders: the gap between theory and practice." *Frontiers in Medicine*. doi: 10.3389/fmed.2018.00285

Kesselheim, A. S., et al. (2015). "Development and use of new therapeutics for rare diseases: views from patients, caregivers, and advocates." *The Patient* 8(1): 75-84.

Kimman, M. L., et al. (2017). "Development and Pretesting of a Questionnaire to Assess Patient Experiences and Satisfaction with Medications (PESaM Questionnaire)." *The Patient* 10(5): 629-642.

Kinder, B. W., et al. (2010). "Predictors for clinical trial participation in the rare lung disease lymphangioleiomyomatosis." *Respiratory Medicine* 104(4): 578-583.

Kuck, C. E., et al. (2016). ""Rare" diseases: Motivated patients make the difference." *International Journal of Cardiology 208: 95-96.*

Ledford, H. (2018). "How Facebook and Twitter could be the next disruptive force in clinical trials." *Nature* 563(7731): 312-315.

Lejbkowicz, I., et al. (2012). "Participatory medicine and patient empowerment towards personalized healthcare in multiple sclerosis." *Expert Review of Neurotherapeutics* 12(3): 343-352.

Li, X., et al. (2020). "The urgent need to empower rare disease organizations in China: an interview-based study." *Orphanet Journal of Rare Diseases.* 15:282

Lopes, M. T., et al. (2018). "Difficulties in the diagnosis and treatment of rare diseases according to the perceptions of patients, relatives and health care professionals." *Clinics.* 73: e68.

Marsh, K., et al. (2019). "Patient Engagement in Clinical Trial Protocol Design and Recruitment Strategies What Does It Mean for Orphan Drug Manufacturers?" Rare Diseases. *The Evidence Forum. White paper.*

Menon, D., et al. (2015). "Involving patients in reducing decision uncertainties around orphan and ultra-orphan drugs: a rare opportunity?" *The Patient* 8(1): 29-39.

Menon, D., et al. (2015). "Developing a patient-directed policy framework for managing orphan and ultra-orphan drugs throughout their lifecycle." *The Patient* 8(1): 103-117.

Miller, P. A., et al. (2016). "Patient-reported outcomes in rare lysosomal storage dieases: Key informant interviews and systematic review protocol." *International Journal of Technology Assessment in Health Care* 32(6): 393-399.

Nicod, E., et al. (2017). "Dealing with Uncertainty and Accounting for Social Value Judgments in Assessments of Orphan Drugs: Evidence from Four European Countries." *Value in Health : the Journal of the International Society for Pharmacoeconomics and Outcomes Research* 20(7): 919-926.

Nicod, E. and P. Kanavos (2016). "Scientific and social value judgments for orphan drugs in health technology assessment." International Journal of Technology Assessment in Health Care 32(4): 218-232.

O'Rourke, P. P. (2013). "Genomic medicine: too great expectations?" *Clinical Pharmacology and Therapeutics* 94(2): 188-190.

Painter, C. A., et al. (2020). "The Angiosarcoma Project: enabling genomic and clinical discoveries in a rare cancer through patient-partnered research." *Nature Medicine* 26(2): 181-187.

Park, J. H., et al. (2020). "How Cancer Patients Perceive Clinical Trials (CTs) in the Era of CTs: Current Perception and Its Differences Between Common and Rare Cancers." *Journal of Cancer Education: the official journal of the American Association for Cancer Education* 35(3): 545-556.

Parsons, C. G. (2019). "CNS repurposing-Potential new uses for old drugs: examples of screens for Alzheimer's disease, Parkinson's disease and spasticity." *Neuropharmacology* 147:4-10

Peay, H. L., et al. (2014). "Expectations and experiences of investigators and parents involved in a clinical trial for Duchenne/Becker muscular dystrophy." *Clinical Trials* 11(1): 77-85.

Perestelo-Perez, L., et al. (2017). "Patient Empowerment and Involvement in Research." *Advances in Experimental Medicine and Biology* 1031: 249-264.

Pogany, G. (2013). "Personalized medicine from the viewpoint of patients and their relatives." *Hungarian Oncology* 57(1): 11-15.

Post, A. E. M., et al. (2021). "Research priorities for rare neurological diseases: a representative view of patient representatives and healthcare professionals from the European Reference Network for Rare Neurological Diseases." *Orphanet Journal of Rare Diseases* 16(1): 135.

Pravettoni, G. and A. Gorini (2011). "A P5 cancer medicine approach: why personalized medicine cannot ignore psychology." *Journal of Evaluation in Clinical Practice* 17(4): 594-596.

Raffai, F. and O. Timmis (2017). "Building the patient community." *Gene Therapy* 24(9): 547-550.

Rheault, M. N., et al. (2020). "The importance of clinician, patient and researcher collaborations in Alport syndrome." *Pediatric Nephrology* 35(5): 733-742.

Rosenberg-Yunger, Z. R. S., et al. (2011). "Priority setting for orphan drugs: an international comparison." *Health Policy* 100(1): 25-34.

Rubinstein, Y. R., et al. (2012). "Informed consent process for patient participation in rare disease registries linked to biorepositories." *Contemporary Clinical Trials* 33(1): 5-11.

Santana, M.-J., et al. (2020). "Patients, clinicians and researchers working together to improve cardiovascular health: a qualitative study of barriers and priorities for patient-oriented research." *BMJ Open* 10(2): e031187.

Saunders, P. A., et al. (2014). "Data sharing for public health research: A qualitative study of industry and academia." *Communication & Medicine* 11(2): 179-187.

Schibeci, R., et al. (1999). "Genetic medicine: an experiment in community-expert interaction." *Journal of Medical Ethics* 25(4): 335-339.

Shalhub, S., et al. (2020). "Assessment of the Information Sources and Interest in Research Collaboration Among Individuals with Vascular Ehlers-Danlos Syndrome." *Annals of Vascular Surgery* 62: 326-334.

Smith, J., et al. (2021). "Lessons from an Experiential Approach to Patient Community Engagement in Rare Disease." *Clinical Therapeutics*. 43 (2): 421-429

Souza, I. P. d., et al. (2019). "A qualitative approach to rare genetic diseases: an integrative revieof the national and international literature." Ciênc. Saúde Coletiva 24 (10): 3683-3700.

Teare, H. J. A., et al. (2017). "The RUDY study: using digital technologies to enable a research partnership." *European Journal of Human Genetics: EJHG* 25(7): 816-822.

Tingley, K., et al. (2021). "Stakeholder perspectives on clinical research related to therapies for rare diseases: therapeutic misconception and the value of research." *Orphanet Journal of Rare Diseases* 16(1): 26.

Tingley, K., et al. (2018). "Using a meta-narrative literature review and focus groups with key stakeholders to identify perceived challenges and solutions for generating robust evidence on the effectiveness of treatments for rare diseases." *Orphanet Journal of Rare Diseases* 13(1): 104.

von Kries, R. and R. Klar (2008). "Rare diseases- from research's to patient's view." *Bundesgesundheitsbl.* 51(5): 479-480.

Wang, R. T. and S. F. Nelson (2015). "What can Duchenne Connect teach us about treating Duchenne muscular dystrophy?" *Current Opinion in Neurology* 28(5): 535-541.

Whitmarsh, I. (2009). "Hyperdiagnostics: postcolonial utopics of race-based biomedicine." *Medical Anthropology* 28(3): 285-315.

Whittal A, Meregaglia M, Nicod E.(2021) The Use of Patient-Reported Outcome Measures in Rare Diseases and Implications for Health Technology Assessment. *Patient* 14(5): 485-503.

Young, A., Menon, D., Street, J., Al-Hertani, W. & Stafinski, T. (2018). Engagement of Canadian Patients with Rare Diseases and Their Families in the Lifecycle of Therapy: A Qualitative Study. *Patient* 11 (3): 353-359.

Young, A., et al. (2018). "A checklist for managed access programmes for reimbursement co-designed by Canadian patients and caregivers." *Health Expectations: an International Journal of Public Participation in Health Care and Health Policy* 21(6): 973-980.

Young, K., et al. (2019). "Patient involvement in medical research: what patients and physicians learn from each other." *Orphanet Journal of Rare Diseases* 14(1): 21.

Ziebland, S. and A. McPherson (2006). "Making sense of qualitative data analysis: an introduction with illustrations from DIPEx (personal experiences of health and illness)." *Medical Education* 40(5): 405-414.
